# Co-localization of autophagy-related protein p62 with cancer stem cell marker dclk1 may hamper dclk1's elimination during colon cancer development and progression

**DOI:** 10.18632/oncotarget.26684

**Published:** 2019-03-22

**Authors:** Badal Chandra Roy, Ishfaq Ahmed, Satish Ramalingam, Venkatakrishna Jala, Bodduluri Haribabu, Prabhu Ramamoorthy, John Ashcraft, Joseph Valentino, Shrikant Anant, Venkatesh Sampath, Shahid Umar

**Affiliations:** ^1^ Departments of Surgery and Cancer Biology, University of Kansas Medical Center, Kansas City, KS, USA; ^2^ Department of Cancer Biology, University of Kansas Medical Center, Kansas City, KS, USA; ^3^ Department of Genetic Engineering, School of Bio-Engineering, SRM Institute of Science and Technology, Kattankulathur, Kanchipuram, Tamil Nadu, India; ^4^ James Graham Brown Cancer Center and Department of Microbiology and Immunology, University of Louisville, Louisville, KY, USA; ^5^ Division of Neonatology, Children's Mercy Hospital, Kansas City, MO, USA

**Keywords:** colon cancer, autophagy, stem cells, dclk1, metastasis

## Abstract

Autophagy may play a critical role in colon cancer stem cells (CCSCs)-related cancer development. Here, we investigate whether accumulation of infection/injury-induced CCSCs due to impaired autophagy influences colon cancer development and progression. When *Apc^++^* mice were infected with *Citrobacter rodentium* (CR; 10^9^CFUs), we discovered presence of autophagosomes with increases in Beclin-1, LC3B and p62 staining during crypt hyperplasia. *Apc1638N/+* mice when infected with CR or subjected to CR+AOM treatment, exhibited increased colon tumorigenesis with elevated levels of Ki-67, β-catenin, EZH2 and CCSC marker Dclk1, respectively. AOM/DSS treatment of *Apc1638N/+* mice phenocopied CR+AOM treatment as colonic tumors exhibited pronounced changes in Ki-67, EZH2 and Dclk1 accompanied by infiltration of F4/80+ macrophages, CD3+ lymphocytes and CD3/β-catenin co-localization. Intestinal and colonic tumors also stained positive for migrating CSC markers CD110 and CDCP1 wherein, colonic tumors additionally exhibited stromal positivity. In tumors from CR-infected, CR+AOM or AOM/DSS-treated *Apc1638N/+* mice and surgically-resected colon tumor/metastatic liver samples, significant accumulation of p62 and it's co-localization with LC3B and Dclk1 was evident. *Apc^Min/+^* mice when infected with CR and *BLT1^−/−^;Apc^Min/+^* mice, exhibited similar co-localization of p62 with LC3B and Dclk1 within the tumors. Studies in HCT116 and SW480 cells further confirmed p62/Dclk1 co-localization and Chloroquin/LPS-induced increases in Dclk1 promoter activity. Thus, co-localization of p62 with Dclk1 may hamper Dclk1's elimination to impact colon cancer development and progression.

## INTRODUCTION

Autophagy is a cellular mechanism for the degradation and recycling of cellular components via the lysosomal pathway and is important for the survival and homeostasis [1]. It is well established that autophagy is also used as a defense mechanism to combat infection of host cells by intracellular pathogens [2, 3]. However, several of these pathogens have developed mechanisms to bypass the autophagic response to promote their survival and proliferation and simultaneously affect the host inflammatory responses. Previous studies have demonstrated that autophagy in intestinal epithelial cells plays an important role in host defense against *Citrobacter rodentium* (CR) infection and the regulation of CR-induced infectious colitis [4]. Investigations in our laboratory have utilized CR-infection-induced murine model that exhibits increased proliferation and an expanded proliferative zone in the mouse distal colon with associated injury or significant histological inflammation depending upon the genetic background [5–10]. We have also established that crypt hyperplasia exhibits both functional and molecular changes typical of the earliest stages of neoplastic transformation [5–10]. Yet, a systematic role for infection or chemical stressors (e.g., AOM or DSS) or a combination thereof, on either autophagy induction or autophagy-related impairment that leads to pro-survival mechanisms culminating in tumorigenesis, has not been thoroughly investigated.

p62 is a multifunctional adapter protein implicated in selective autophagy, cell signaling pathways and tumorigenesis [11]. Autophagy is activated in cancer cells in response to various anticancer therapies and modulation of p62 stability by autophagy can play diverse roles in tumorigenesis [11]. In addition, p62 has been labeled as a major pro-oncogenic regulator of several important signaling pathways including NF-κB, p44/42 MAPK and mTOR [12, 13]. Cancer stem cells (CSCs) have special capacities for self-renewal, differentiation and tumor formation and are regarded as the major cause of failure in anticancer therapy, such as chemo-and/or radioresistance, tumor recurrence and metastasis [12, 14]. Autophagy may play a dual role in CSCs-related resistance to anticancer therapy [15]. However, the contribution of autophagy in the physiology of CSCs appears complex and is not yet fully elucidated. In the current report, we examined the components of the autophagic machinery and a probable link between autophagy and cancer stemness based on the hypothesis that infection and/or injury-related alterations in autophagy may hamper CSCs elimination which may lead to colon cancer development and/or progression.

## RESULTS

**Figure 1:**
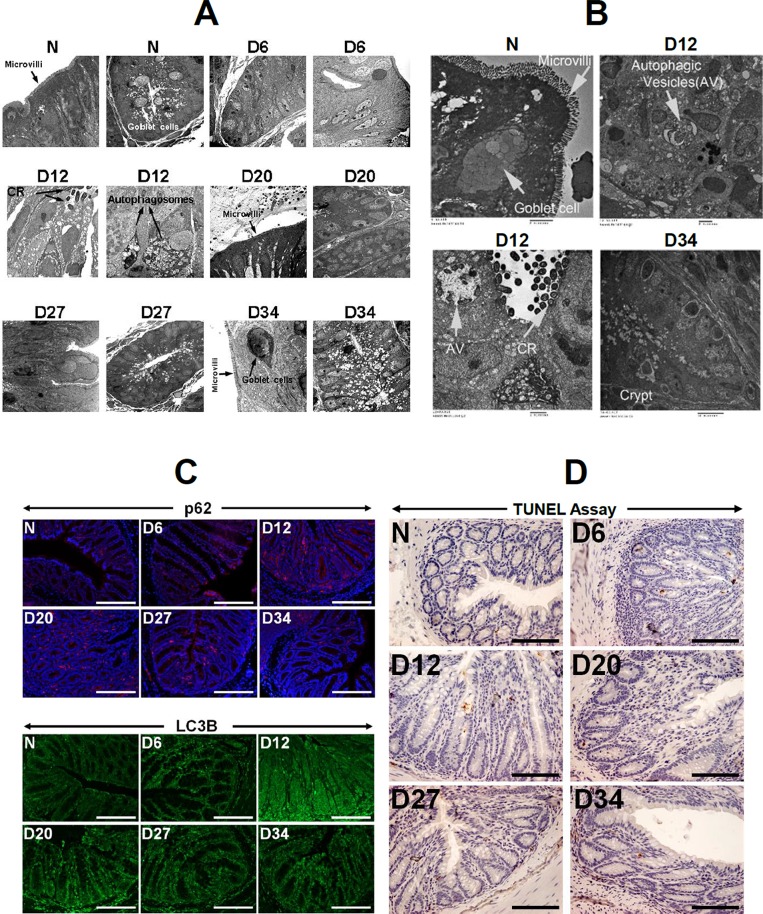
Evidence of basal autophagy in response to CR infection **(A** and **B)** Electron microscopy in the distal colons of uninfected (N) or mice infected with CR at days 6, 12, 20, 27 and 34, showing presence of CR at the luminal surface and autophagosomes/autophagic vesicles AV; arrows). Both microvilli and goblet cells can also be seen. **(C)** Immunofluorescent staining of N-D34 samples with antibodies for p62 and LC3B, respectively. Scale bar = 150μm; n = 3 independent experiments. **(D)** Immunohistochemical labeling of TUNEL positive cells within the crypt of N-D34 mouse distal colons. Scale bar = 100μm; n = 3 independent experiments.

Previous studies have demonstrated that autophagy in intestinal epithelial cells plays an important role in host defense against *Citrobacter rodentium* (CR) infection and in the regulation of infectious colitis [4]. However, the mechanistic role of CR-induced autophagy in regulating cellular proliferation and neoplastic transformation underlying tumor development is not well understood. In response to CR infection, electron microscopy revealed significant increase in autophagic vacuolation and autophagic vesicles were widespread at 12 days post-infection in the *Apc++* NIH:Swiss (Figure 1A, 1B). During measurement of Beclin-1, LC3B and p62 kinetics in *Apc++* NIH:Swiss mice, the staining intensities for these proteins increased significantly at day 12 and subsequently declined at days 20, 27 and 34 post-infection (Figure 1C; Supplementary Figure 1A). In addition to NIH:Swiss outbred mice, colons of C57Bl/6 inbred mice also exhibited elevated levels of Beclin-1, LC3B and p62 staining in response to CR infection (Supplementary Figure 1B-1D). The kinetics for Beclin-1 and LC-3B were similar to NIH:Swiss with peak responses between 9-12 days while p62 remained elevated even at day 19 post-infection (Supplementary Figure 1D). Interestingly, TUNEL assay, a measure of apoptosis, did not reveal any significant change in response to infection (Figure 1D) suggesting that the apoptotic pathway may not be underpinning CR pathogenesis.

**Figure 2:**
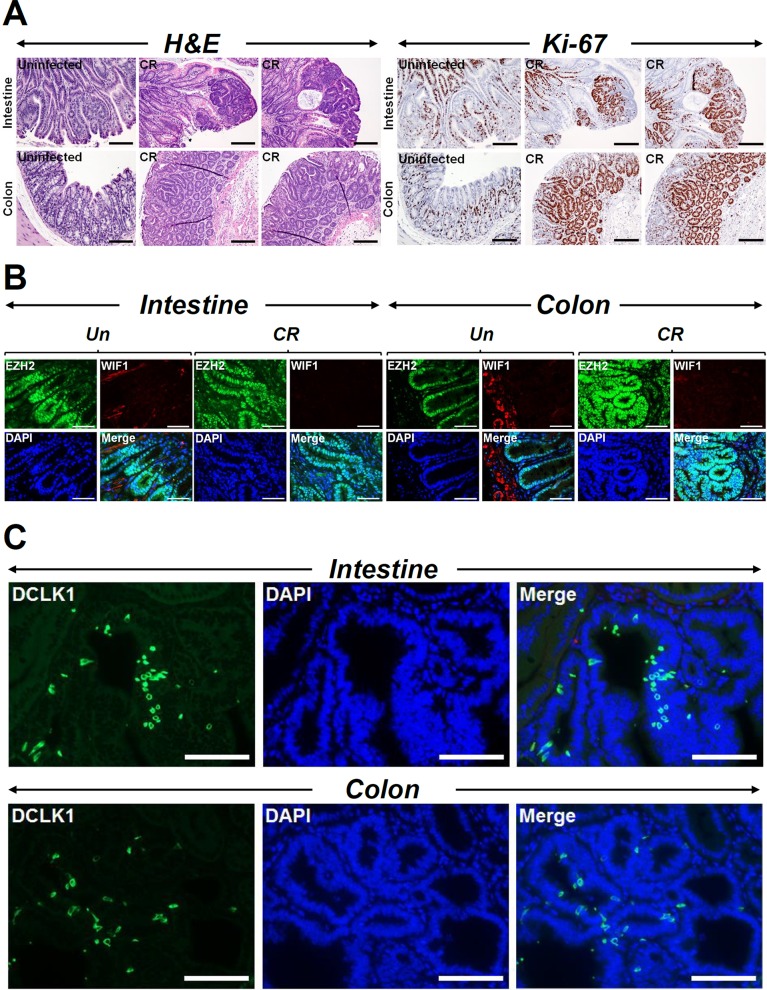
CR infection influences tumorigenesis **(A)** Paraffin-embedded sections prepared from the intestinal or colonic tumors isolated from uninfected (N) or CR infected Apc1638N/+ mice were subjected to H&E staining for gross morphology or Ki-67 staining to detect cell proliferation (Scale bar = 75 μm; n = 3 independent experiments). **(B** and **C)** Immunofluorescent staining of uninfected (Un) or CR infected (CR) intestinal or colonic tumors with antibodies for EZH2 and WIF-1 **(B)** and Dclk1 **(C)**. Scale bars = 150μm (b); 250μm (c); n = 3 independent experiments.

Since dysregulation of autophagy is not only associated with microbial infections but with numerous disease manifestations including cancer, we began to systematically look at murine models of colon cancer and whether autophagy played a role in tumor development and/or progression. Using the *Apc1638N/+* model that mimics familial adenomatous polyposis, we observed CR to minimally impact tumorigenesis in the small intestine as is revealed in Figure 2 consistent with CR being a colonic pathogen. Indeed, in response to CR infection, colons displayed significant increase in tumor development at 90-120 days post-infection and these tumors were highly proliferative as was revealed by Ki-67 staining (Figure 2A). Interestingly, both intestinal and colonic tumors exhibited elevated levels of EZH2 (Figure 2B) and β-catenin (Supplementary Figure 1E) staining, respectively. WIF-1 staining on the other hand, while positive in untreated intestine/colon sections, were undetectable in CR-treated tumors (Figure 2B). These results are consistent with our previous reports [10]. Since cancer stem cells (CSCs), which comprise a small proportion of total cancer cells, have special capacities for self-renewal, differentiation and tumor formation and Dclk1 is a validated marker of CSCs, we next stained tumors from either organ with antibody for Dclk1. As is revealed in Figure 2C and Supplementary Figure 2, we discovered dramatic increases in Dclk1 staining in sections prepared from both intestinal and colonic tumors consistent with Dclk1's role in tumor development [16, 17].

**Figure 3:**
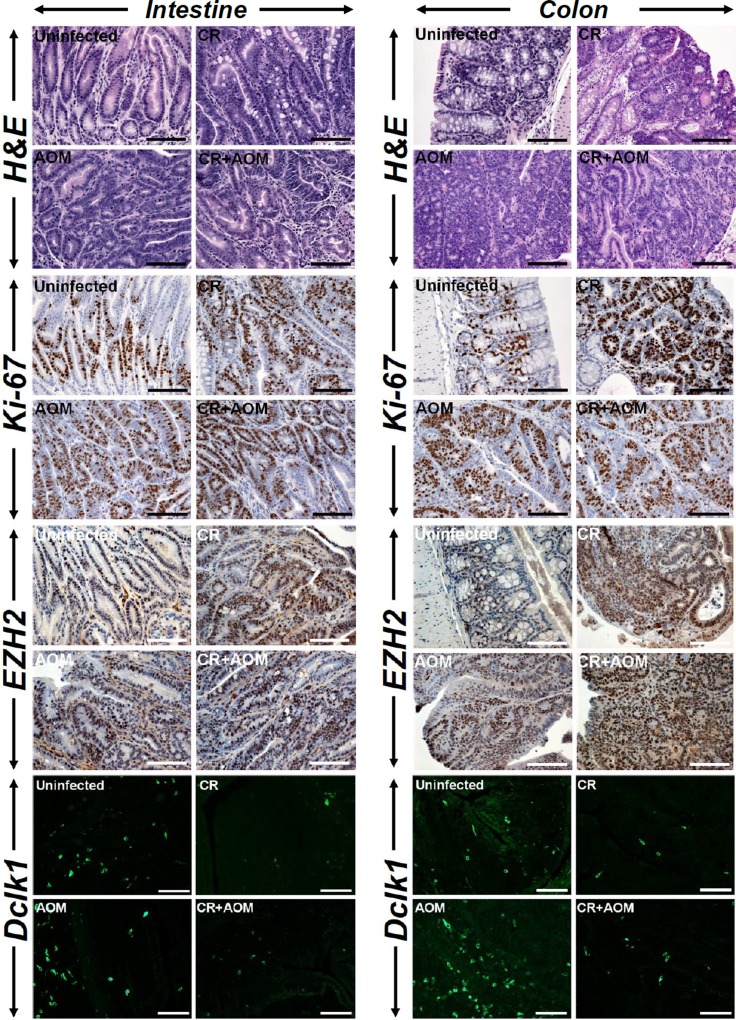
Measuring the combined effect of infection and mutagenic injury on tumorigenesis Paraffin-embedded sections prepared from the intestinal or colonic tumors isolated from uninfected (Un), CR infected (CR), AOM-treated or CR+AOM-treated *Apc1638N/+* mice were subjected to H&E staining for gross morphology or Ki-67 staining to detect cell proliferation. Sections were also immuno-stained with antibodies for EZH2 and Dclk1, respectively. DAPI was used as nuclear stain. Scale bars = 150-250μm; n = 3 independent experiments.

Since *Apc1638N/+* mice have previously been shown to exhibit metastatic potential [13], we next investigated the probability of a mutagenic insult when combined with CR infection to expedite tumor progression. As is revealed in Figure 3, CR+AOM treatment did not necessarily have an additive effect on tumorigenesis in either the intestine or the colon compared to CR infection alone although the staining intensities for Ki-67 and EZH2 were higher in CR, AOM and CR+AOM groups compared to untreated controls. Interestingly, AOM treatment alone induced significant Dclk1 staining in the intestinal tumors and more so in the colon. Since Dclk1 as a microtubule regulator marks a morphologically distinct and functionally unique population of cancer-initiating cells with molecular features of gastrointestinal tuft cells [18], we co-stained the sections with antibodies for Acetylated Tubulin (AcTub) and Dclk1, respectively. As is revealed in Supplementary Figure 3A, the staining for AcTub was virtually identical with that of Dclk1 suggesting Dclk1/AcTub to represent the CSC population. These results underscore the complexity of tumorigenesis in *Apc1638N/+* mice wherein, both infection and chemical stressors are equally capable of inducing CSCs.

**Figure 4:**
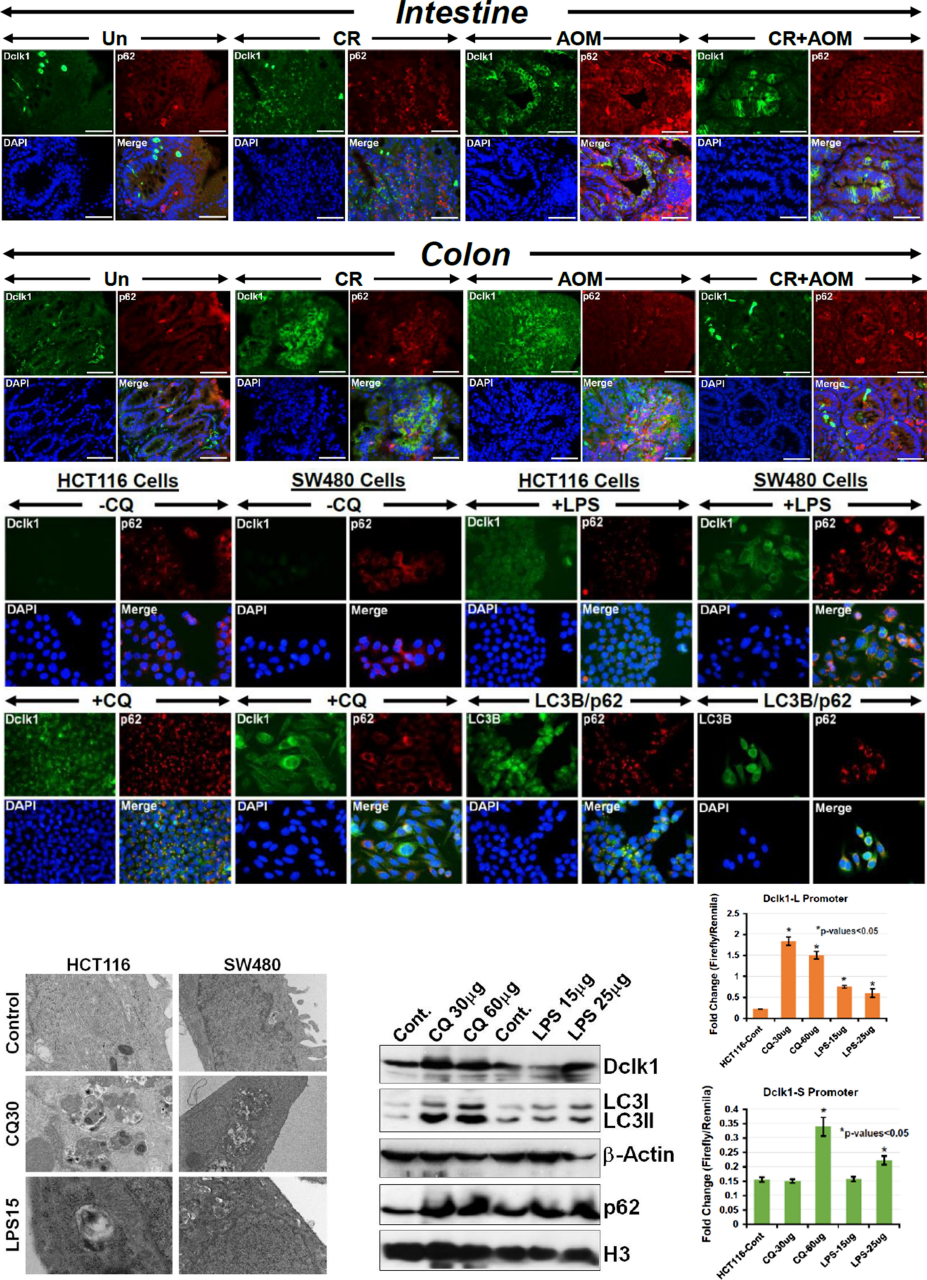
Evidence of altered autophagy in the intestinal/colon tumors Paraffin-embedded sections prepared from the intestinal or colonic (upper panels) tumors isolated from uninfected (Un), CR infected (CR), AOM-treated or CR+AOM-treated *Apc1638N/+* mice were subjected to co-localization studies with antibodies for p62 and Dclk1, respectively. DAPI was used as nuclear stain. Co-localization of p62 and Dclk1 appears as yellow/orange puncta in the merged images. Scale bars = 150-250μm; n = 3 independent experiments. Middle panel. *In vitro* evidence of autophagy flux.HCT116 and SW480 cells treated for 24hr with either Chloroquin (CQ, 30μM) to block autophagy and promote efficient autophagic flux or LPS to mimic CR infection. Cells were processed and co-localization of p62 with Dclk1 and p62 with LC3B was evaluated. DAPI was used as nuclear stain (n = 3 independent experiments). Co-localization of p62 and Dclk1 appears as yellow/orange puncta in the merged images. Lower panel. Left:Presence or absence of autophagic vesicles was further confirmed via electron microscopy wherein, the effect of both autophagy inhibitor CQ or LPS was examined (n = 3 independent experiments). Middle: Western blotting revealed a dose-dependent increase in Dclk1, LC3-II and p62, respectively. β-Actin and H3 were used as loading controls. Right: Dclk1 promoter assay. HCT116 cells transfected with Dclk1-L or Dclk1-S reporters were either untreated or treated with varying dosages of CQ or LPS followed by measurement of reporter activity (^*^p-values<0.05 vs control; n=3 independent experiments).

Since autophagy represents a pro-survival mechanism implemented by neoplastic cells in response to stressful stimuli and Dclk1's expression as a marker of CCSCs, is associated with cancer growth, EMT and tumor metastasis, we next explored probable interaction between p62 and Dclk1+ CCSCs *in vivo* and in cell lines. As is depicted in Figure 4, both intestinal and colonic tumors exhibited significant co-localization of Dclk1 with p62. Colonic tumors in particular, responded positively to both CR and CR+AOM wherein, Dclk1 and p62 co-localization was clearly evident (Figure 4, Intestine & Colon panels). As proof of concept, we utilized two validated colon cancer cell lines to confirm Dclk1's co-localization with p62. In both HCT116 and SW480 cells treated with either CQ (30μM) to block autophagy and promote efficient autophagic flux or LPS to mimic *Citrobacter rodentium* (CR) infection, we discovered significant co-localization of Dclk1 with p62 compared to untreated controls (Figure 4, middle panels). Since the level of p62 can also be modulated independent of autophagy, we employed additional methods to assess autophagic flux wherein we monitored LC3 turnover [19, 20]. In the presence of CQ, LC3-II accumulated indicating efficient autophagic flux and co-localized with p62 (Figure 4, middle, lower right panels). The enhanced accumulation of autophagic vesicles in the presence of either CQ or LPS was further confirmed by electron microscopy (Figure 4, lower left panel). The expression levels of LC3-I/II, p62 and Dclk1 in HCT116 cells when treated with CQ and LPS increased dose dependently as was revealed by Western blotting (Figure 4, lower middle panels). Since long (Dclk1-L) and short (Dclk1-S) isoforms of Dclk1, regulated by α- and β-promoters, have been recently described [21], we next cloned the two promoters and performed a promoter-reporter activity. As is shown in Figure 4, both CQ and LPS significantly increased the Dclk1-L reporter activity independently of their dosages. Interestingly, CQ at 30μg failed to influence Dclk1-S activity. At 60μg however, a >2.0-fold increase in reporter activity was recorded. Similar responses were seen with varying dosages of LPS (Figure 4, graphs, lower right). Since there is an unmet need to breed either *Apc1638N/+* or *Apc^Min/+^* mice with *Beclin-1* or *Atg7* knockout mice to definitively assess the impact of autophagy on colon cancer development, for the current study, we investigated the effect of Atg7 or Beclin-1 knockdown on Dclk1 promoter-reporter activity. As is depicted in Supplementary Figure 3B and 3C, siRNAs to either ATG7 or Beclin-1: i) reduced ATG7 and Beclin-1 protein levels (Supplementary Figure 3B) and ii) significantly inhibited Dclk1 reporter activity without necessarily impacting Dclk1 protein levels (Supplementary Figure 3C, 3B) suggesting that autophagy-related proteins and Dclk1 may functionally interact to influence Dclk1's CSC capabilities.

**Figure 5:**
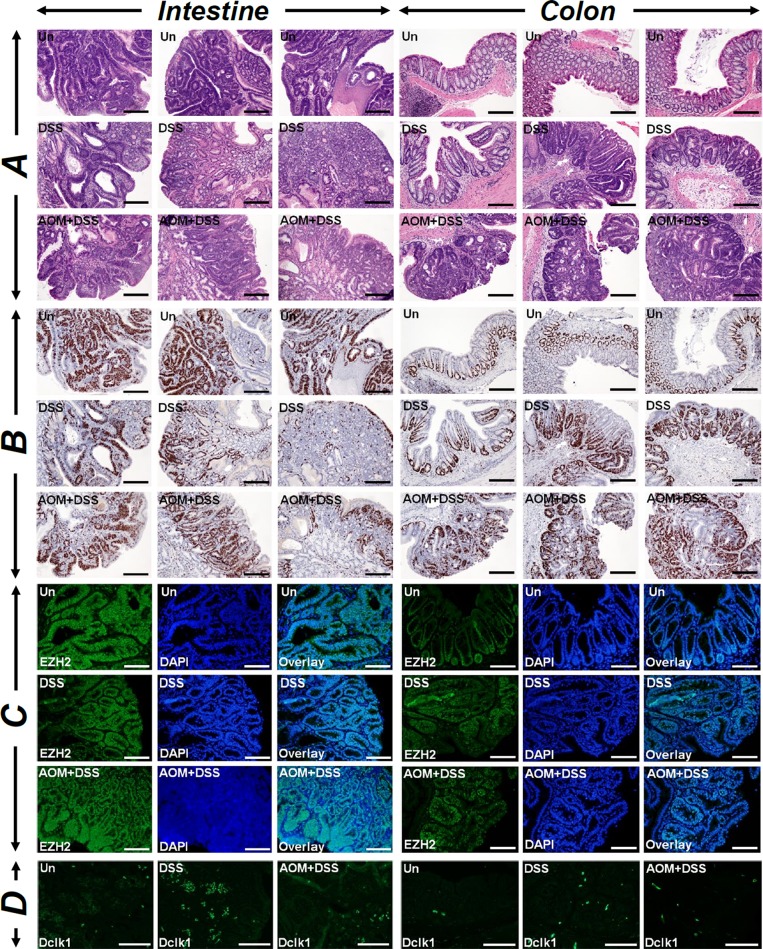
Measuring the combined effect of DSS and AOM+DSS on tumorigenesis Paraffin-embedded sections prepared from the intestinal or colonic tumors isolated from untreated (Un), DSS-treated or AOM+DSS-treated *Apc1638N/+* mice were subjected to H&E **(A)**, Ki-67 **(B)**, EZH2 **(C)** and Dclk1 **(D)** staining, respectively. A montage of three representative images for each group is shown. DAPI was used as nuclear stain. Scale bars = 150-250μm; n = 3 independent experiments.

**Figure 6:**
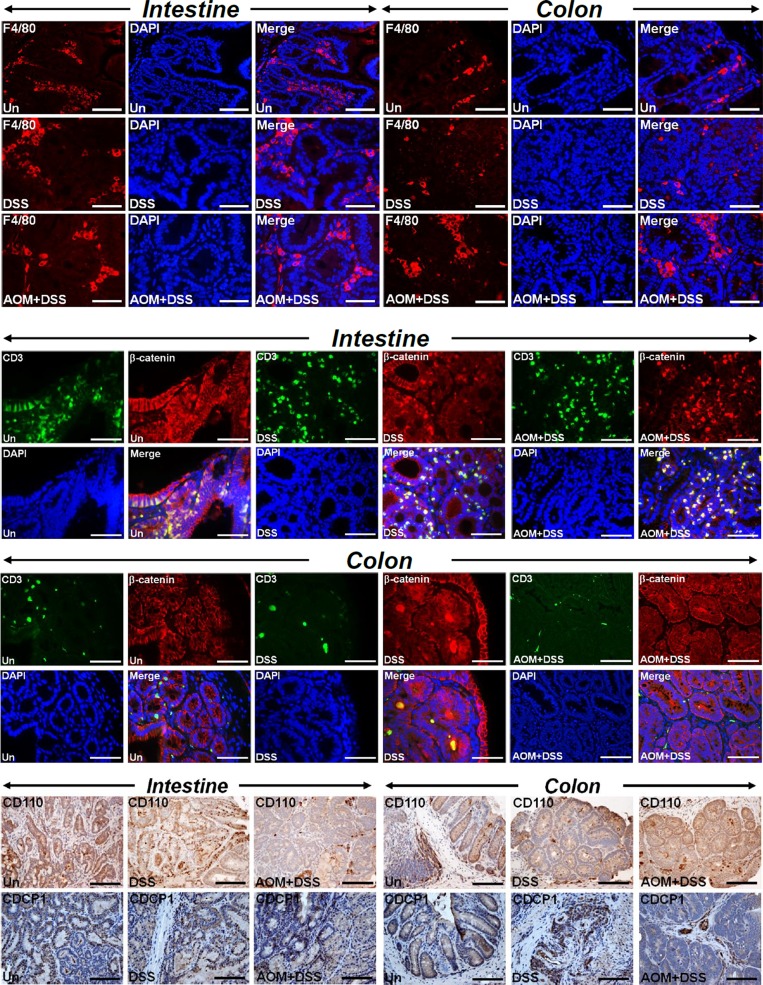
Recruitment of immune cells and evidence of local spread Paraffin-embedded sections prepared from the intestinal or colonic tumors isolated from untreated (Un), DSS-treated or AOM+DSS-treated *Apc1638N/+* mice were subjected to F4/80 (upper panel), CD3/β-catenin co-localization (middle panel) and CD110 and CDCP1 (lower panel) staining, respectively. DAPI was used as nuclear stain. Scale bars = 150-250μm; n = 3 independent experiments.

Chronic intestinal inflammation is a risk factor for development of colon cancer and azoxymethane (AOM) and dextran sodium sulfate (DSS) combination has been shown to model colitis-associated colon cancer. Since defects in autophagy have been reported in inflammatory bowel diseases and p62 has been shown to be upregulated in ulcerative colitis patients [22], we wanted to investigate if CR/AOM-induced changes in autophagy could be phenocopied in *Apc1638N/+* mice receiving AOM/DSS. As is depicted in Figure 5, both small intestine and colon tumors responded very aggressively to either DSS or AOM/DSS with colonic tumors exhibiting gradual increases in Ki-67, EZH2 and Dclk1 staining in treated samples compared to untreated controls. Dclk1 again co-stained with AcTub (Supplementary Figure 4). These tumors were also highly inflamed as was revealed by infiltration of F4/80+ macrophages (Figure 6, upper panel) and CD3+ lymphocytes (Figure 6, upper middle panel). Interestingly, CD3 co-localized with β-catenin in these tumors indicating contribution of Wnt signaling in the tumorigenic process (Figure 6, middle two panels). Both intestinal and colonic tumors also stained positive for migrating cancer stem cell markers CD110 and CDCP1 (Figure 6, lower panel) and Lgr5 (Supplementary Figure 5), respectively. Colonic tumors additionally exhibited stromal positivity suggesting local spread (Figure 6, lower panel).

**Figure 7:**
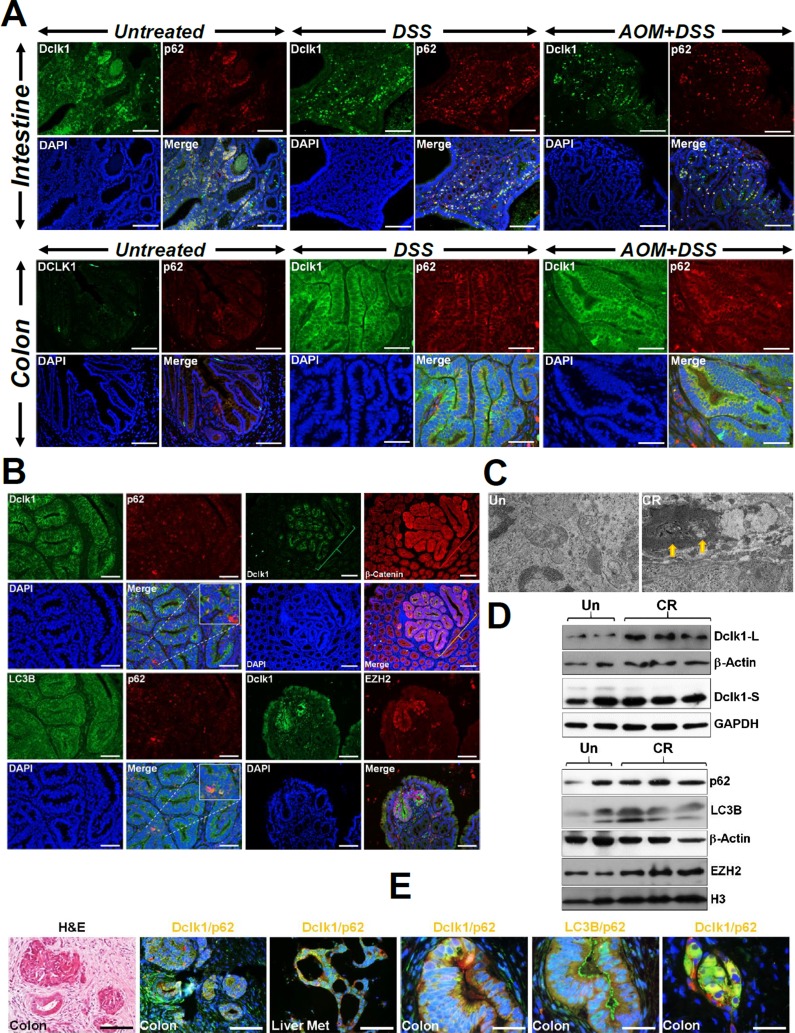
Epithelial injury promotes accumulation of p62 Paraffin-embedded sections prepared from the intestinal or colonic tumors isolated from untreated (Un), DSS-treated or AOM+DSS-treated *Apc1638N/+* **(A)** or CR-infected *Apc^Min/+^* **(B)** mice were co-stained with antibodies for p62 and Dclk1 **(A, B)**, Dclk1 and β-catenin or Dclk1 and EZH2, respectively **(B)**. Inset in B shows merged images of the p62/Dclk1 puncta. Scale bars = 150-250μm; n = 3 independent experiments. **(C)** Electron microscopy showing presence of autophagic vesicles in the CR-infected but not uninfected *Apc^Min/+^* mouse distal colonic fragments (2μm sections; arrows; n = 3 independent experiments). **(D)** Western blotting to detect changes in Dclk1, p62, LC3B and EZH2, respectively. GAPDH and β-Actin were used as loading controls. **(E)** Evidence of p62 and Dclk1 co-localization in surgically resected colon cancer samples with metastasis to the liver. Paraffin-embedded sections prepared from surgically resected colon tumors and liver mets were co-stained with antibodies for p62 and Dclk1 and p62 and LC3B, respectively. Representative merged images are shown with an H&E stained section showing the gross morphology. Scale bars = 450μm.

Further investigation of tumors from AOM/DSS-treated *Apc1638N/+* mice revealed significant colocalization of p62 with Dclk1 (Figure 7A). To ensure that changes in p62 were indeed due to autophagy, we examined co-localization of p62 with LC3B. As expected, the staining for p62 overlapped with that of LC3B in both the intestinal and the colonic tumors, respectively (Supplementary Figure 6A). We also confirmed presence of autophagic vesicles within the tumors via electron microscopy (Supplementary Figure 6B) and relative levels of Dclk1, p62 and LC3B via Western blotting (Supplementary Figure 6C). These findings were further validated in *ApcMin/+* mice infected with CR (Figure 7B) wherein, Dclk1/p62 co-localization was evident along with Dclk1's co-localization with β-catenin and EZH2. Presence of autophagic vesicles was confirmed via electron microscopy (Figure 7C) and relative levels of Dclk1, p62 and LC3-I/II were validated through Western blotting (Figure 7D). In *BLT1^−/−^;Apc^Min/+^* mice that exhibit exaggerated tumorigenesis in the intestine and particularly in the colon ^10^, we found similar increases in Dclk1 and its co-localization with both Atg7 and p62, respectively (Supplementary Figure 7A). Since inflammation drives the tumorigenic process in these mice, we also stained sections from these mice with antibody for CD3. As is depicted in Supplementary Figure 7B, we discovered increases in CD3 and its co-localization with β-catenin similar to the staining pattern seen with AOM/DSS (see Figure 6). Changes in Dclk1, p62, LC3-I/II and EZH2 were further validated via Western blotting (Supplementary Figure 7C).

Prior studies have shown that a subset of Dclk1-positive CSCs survive due to autophagy in response to curcumin [23] and that increased Dclk1 correlates with the malignant status and poor outcome in malignant tumors [24]. We next examined surgical samples from a patient with multiple tumors in the colon and metastasis to the liver. As is shown in Figure 7E and Supplementary Figure 7D and 7E, both the colon tumors and liver mets exhibited significant co-localization of Dclk1 with p62 suggesting that p62/Dclk1 interaction may be relevant in the pathogenesis of tumor development and progression.

## DISCUSSION

Autophagy, a fundamental cellular process important for survival and homeostasis, is also used as a defense mechanism to combat infection [2]. Pathogens have however developed mechanisms to avoid the autophagic response to prolong their survival. Previous studies have demonstrated that autophagy in intestinal epithelial cells plays an important role in host defense against *Citrobacter rodentium* (CR) infection and the regulation of CR-induced infectious colitis [4]. In this study, we provide evidence that CR infection induces basal autophagy. Moreover, CR infection when combined with AOM exhibit significant increase in Dclk1+ CCSCs that co-localizes with autophagy-related protein p62. We further provide evidence of p62/Dclk1 interaction in AOM/DSS and *BLT1^−/−^;Apc^Min/+^* models of colon cancer as well as in surgical samples of a colon cancer patient with liver metastasis. These findings strongly suggest that p62/Dclk1 interaction may contribute towards tumor development and progression in the colon.

Autophagy plays a significant role in controlling the pathogenesis of infectious diseases by regulating processes related to pathogen clearance, lymphocyte development, antigen presentation and immunoglobulin production etc., [2, 3]. Impaired autophagy contributes to oxidative stress, genomic instability, chronic tissue damage, inflammation and tumorigenesis, and is involved in aberrant bacterial clearance and immune priming. *Salmonella Typhimurium* for example, interferes with host responses via delivery of the effector proteins into cytosol to promote its survival and proliferation [3]. We demonstrate significant induction of basal autophagy with accumulation of Beclin-1, LC3B and p62 at peak crypt hyperplasia. Interestingly, there was no parallel increase in apoptosis of crypt epithelial cells. In a recent study, it was revealed that activation of autophagy results in inhibition of apoptosis [25]. Similarly, ectopic overexpression of Cdx1 in CD44+CD24+p53wt stem cells induced LC3-II and inhibited cell death from paclitaxel treatment suggesting that activation of autophagy inhibited apoptosis [26]. Since we have shown previously that activation of Wnt/β-catenin, Notch and NF-κB pathways regulate CR-induced crypt hyperplasia [5–10, 27, 28], it is tempting to speculate that induction of basal autophagy in response to CR infection is required to inhibit apoptosis and to promote crypt hyperplasia reminiscent of earliest stages of neoplastic changes associated with colon cancer [9, 10]. This speculation is not unprecedented since an elegant study recently showed that *Fusobacterium nucleatum*-induced autophagy inhibited apoptosis leading to recurrence of colorectal cancer and poor outcomes [29].

Colon cancer stem cells have been identified as one of the major contributors to resistance of colon cancer to chemotherapy [30]. Doublecortin-like kinase 1 (Dclk1) is a validated CSC marker in the gastrointestinal tract [18, 31]. We have previously shown significant changes in Dclk1 expression during crypt hyperplasia and tumorigenesis [9]. Using various models of colon carcinogenesis, we now present evidence of significant upregulation of Dclk1 within the tumors and that Dclk1 co-localizes with p62. Aberrant autophagy associated with p62 dysfunction is involved in the pathogenesis of human diseases. Indeed, aberrant accumulation of p62-positive aggregated structures has been detected in patients with liver disorders, tumors and neurodegenerative diseases [11, 32]. p62 is also a pathogenic target of 5q copy number gains in kidney cancer [33]. In colon cancer patients, a positive correlation has been reported between tumor progression and p62 expression [34] in accordance with p62's role as a significant contributor to tumorigenesis [35]. Interestingly, p62 levels positively correlated with Wnt regulator Dvl2 in late stage tumors indicating that impairment of autophagy may contribute towards aberrant Wnt activation [34]. Similarly, stimulation of tumor growth by p62 accumulation in FIP200 (FAK family-interacting protein of 200 kDa)-null tumors was associated with the activation of NF-κB pathway [36]. Coincidentally, we have previously reported activation of both Wnt and NF-κB pathways in models of CR-induced crypt hyperplasia and tumorigenesis (5-10, 15, 16). Thus, Dclk1 accumulation and its co-localization with p62 that we see within the tumors does not seem to be merely a histologic marker. Yet, whether it directly contributes towards tumor growth and progression is being elucidated in colon-specific Dclk1 knockouts as they are being bred to *ApcMin/+* mice to definitively link Dclk1 in colon cancer development and progression in response to CR infection.

Intriguingly, p62 seems to co-localize with Dclk1 as a punctate staining at the plasma membrane while occasional co-localization could be seen within the nucleus. Since Dclk1 exists in a membrane-bound long (Dclk1-L) and membrane/nuclear localized short (Dclk1-S) forms consistent with presence of both transcripts coded for by α- and β-promoters respectively [24], our findings suggest that both isoforms of Dclk1 are probably involved in the tumorigenic process. The increases in Dclk1-L and Dclk1-S promoter activities following treatment with either autophagy inhibitor CQ or LPS further support our conclusion. Finally, staining of the surgically resected tumors from patients with colon cancer and metastasis to the liver clearly demonstrated co-localization of p62/Dclk1 puncta both at the plasma membrane and within the nucleus suggesting involvement of both isoforms. In an elegant study, Kantara et al., [23] previously described that a subset of Dclk1-positive CSCs survive due to autophagy in response to curcumin. In line with this observation, our findings provide a more comprehensive look into the complexity of Dclk1 regulation during tumorigenesis and suggest that Dclk1+ cells may survive due to impaired autophagy. Since it is generally believed that autophagy contributes to stemness maintenance of CSCs [15] and increases in Dclk1 correlates with the malignant status and poor outcome in malignant tumors [24], our studies provide a rationale to capture these interactions early through small molecule inhibitor-based Dclk1 targeting to block Dclk1 from interacting with p62 as a strategy to prevent or treat colon cancer.

## MATERIALS AND METHODS

### Animals

NIH:Swiss, C57Bl/6 and *Apc^Min/+^* mice were procured from Envigo and Jackson Laboratory, respectively. *Apc1638N/+* mice were initially acquired from NCI Repository and subsequently bred in-house to reach the numbers needed for each experiment. *BLT^−/−^; Apc^Min+^* mice were generated as described [10]. All the mice were maintained in a specific pathogen-free (including helicobacter and parvovirus) environment and generally used between 5 and 6 weeks of age. As control groups, either littermates or WT mice of identical background were used. This study was carried out in strict accordance with the recommendations in the Guide for the Care and Use of Laboratory Animals of the National Institutes of Health. All protocols were approved by the Institutional Animal Care and Use Committee of the University of Kansas Medical Center.

### Treatments

Colonic crypt cell hyperplasia was induced in the mice by oral inoculation with a 16-h culture of *C. rodentium* (biotype 4280, ATCC, 10^8^CFUs) identified as pink colonies on MacConkey agar, as previously described [5–10, 27, 28]. Biotype 4280 is a unique mouse-specific strain that adheres to mature surface colonocytes within the distal colon to induce histopathological changes known as attaching and effacing lesions [37]. Adherent bacteria were assayed using RT-PCR for bacterial intimin in whole tissue extracts [38]. Age- and sex-matched control mice received sterile culture medium only. For AOM and DSS studies, 5-6 weeks old *Apc1638N/+* mice on day 0 were injected intraperitoneally (IP) with 10 mg/kg of AOM working solution (1 mg/ml in isotonic saline, diluted from 10 mg/ml stock solution in dH2O kept at −20 °C). A 2.5% (2.5 g/100 ml) DSS solution in distilled water was readied and passed through a 0.22 μm cellulose acetate filter by vacuum. On day 7, DSS solution was supplied to mice in their drinking water. Approximately 250 ml/cage was needed every time new DSS was provided for a maximum of 5 mice/cage. To provide a continuous supply of DSS for seven days, DSS solution was replaced in clean bottles three times (every 2-3 days) during this period. On day 14, cages were switched back to standard drinking water for the duration of the experiment. All untreated or treated mice were euthanized at specified time points. To examine the presence or absence of autophagy flux *in vitro*, we used HCT116 and SW480 colon cancer cells and treated them with either Chloroquin (CQ; 30 or 60μM) or LPS (15 or 25μg/ml) (Sigma-Aldrich, St. Louis, MO, USA) for 24hrs followed by measurement of autophagy-related markers via immunostaining and Western blotting.

### Histology

Colon tissues/tumors were freshly harvested from mice and fixed with 10% neutral buffered formalin prior to paraffin embedding. De-identified and surgically resected colon tumors and liver mets were likewise fixed in 10% neutral buffered formalin prior to paraffin embedding. Paraffin-embedded sections (4 μm) were stained with Hematoxylin and Eosin for morphology using standard techniques. Goblet cells were stained with PAS (Richard-Allan Scientific) or Alcian blue (Thermo Scientific) and counterstained with Nuclear Fast Red (Sigma-Aldrich). The pictures were obtained with a Nikon i80 microscope (Nikon Instruments, Melville, NY, USA).

### Immunohistochemistry

Paraffin embedded sections were dewaxed with xylene and rehydrated in graded series of ethanol. Tissue sections were then washed with PBS before starting staining procedure. Immunohistochemistry was done using Histostain-SP kit (Invitrogen, Carlsbad, CA, USA) and following their instructions. Briefly, deparaffinized sections were boiled for 20 minutes in 0.01 M citrate buffer at pH 6.0 for epitope retrieval. The sections were then blocked for 10 minutes using a serum blocking solution. Sections were incubated for 1 hour at 4°C with primary antibodies (described below). After 3 washings of 5 minute each in PBS, sections were subsequently incubated with biotinylated secondary antibodies for 10 minutes, followed by 10-minute incubation with enzyme streptavidin-peroxidase conjugate. This was followed by 3-5 minute incubation with DAB solution containing diaminobenzidine (DAB) chromogen for developing and 0.6% H_2_O_2_ to inhibit endogenous peroxidase activity. The sections were finally counterstained with hematoxylin and dehydrated with graded series of ethanol and cleared in xylene. After staining, the sections were mounted with mounting medium and images were obtained and analyzed with a Nikon i80 microscope.

### Immunoflourescence

After dewaxing and rehydration, tissue sections were incubated with relevant primary antibody overnight at 4°C. Antibodies used were rabbit anti-DCLK1 (1:200, ab31704, ab109029) from Abcam (Cambridge, UK), mouse anti-p62/SQSTM1 (1:300, H00008878-M01), rabbit anti-Beclin1 (1:200, NB500-249), anti-LC3B (1:250, NB100-2220) from Novus Biologicals (Littleton, CO, USA). Anti-CDCP1 from Boster, (Pleasanton, CA, USA), CD110 from LSBio, (Seattle, WA, USA) and Anti-F4/80 (Thermo Fisher). Anti-β-catenin, ant-CD3 from BD Biosciences (San Jose, CA, USA), anti-EZH2, anti-H3 were from Cell Signaling Technology (Danvers, MA, USA) and anti-WIF1 from Santa Cruz Technology (Santa Cruz, CA, USA). After incubation, the slides were washed 3 times in PBS for 5 min each. The secondary antibody staining was performed by covering the tissue sections with a 1:1000 dilution of Alexa Fluor 488 / 594 conjugated goat anti-rabbit / anti-mouse IgG antibody (original concentration: 2 mg/ml; (Thermo Fisher Scientific, Waltham, MA, USA) in blocking buffer and the sections were incubated for 1 hour at room temperature in dark. The excess liquid was blotted away and the sections were rinsed twice for 5 min each using PBS. Next, the sections were stained for 5 min at room temperature in dark using a 10 mg/ml of DAPI solution diluted in 1x PBS (Sigma-Aldrich, St. Louis, MO, USA). The sections were then rinsed with Milli-Q water and blotted dry. Finally, the sections were covered with ProLong Gold Antifade Mounting media (Invitrogen, Carlsbad, CA, USA), covered with coverslips and the edges of the coverslips sealed with nail polish. The slides were kept at room temperature in dark for at least 24 h and then visualized by Nikon i80 upright fluorescence microscope.

### Quantitative Reverse-Transcriptase PCR

RNA was isolated using TRIzol (Life Technologies, Grand Island, NY, USA) and converted to cDNA using the High-Capacity cDNA Reverse Transcription kit (Applied Biosystems, Foster City, CA, USA). The concentration of RNA was measured using a spectrophotometer (Nanodrop 2000, Thermo Fisher Scientific). Gene expression was assessed using Jumpstart Taq Polymerase (Sigma-Aldrich) and SYBR Green nucleic acid stain (Life Technologies). Threshold crossing values for each gene were normalized to glyceraldehyde phosphate dehydrogenase (GAPDH) gene expression. mRNA expression was then normalized to fold change relative to controls.

### Western blotting

Total crypt cellular or nuclear extracts (30–50 μg protein/lane), were subjected to SDS-PAGE and electrotransferred to nitrocellulose membrane. The membranes were blocked with 5% BSA or 5% nonfat dried milk in Tris-buffered saline (TBS) (20 mM Tris-HCl and 137 mM NaCl, pH 7.5) for 1 h at room temperature (21 °C). Immunoantigenicity was detected by incubating the membranes overnight with the appropriate primary antibodies (0.5-1.0 μg/ml in 5% BSA or 5% nonfat dried milk) and Western blot analysis was performed as described [10]. Antibodies used were anti-DCLK1 (1:1000, ab31704, ab109029) from Abcam, anti-p62/SQSTM1 (1:400, H00008878-M01), anti-LC3B (1:300, NB100-2220) from Novus Biologicals, anti-GAPDH(1:3000), anti-β-actin (1:5000), from Sigma-Aldrich, anti-EZH2 (1:000), anti-H3 (1:1000) were from Cell Signaling Technology.

### Statistical analyses

Experiments were repeated three times with consistent results. Data were expressed as mean values ± standard error. Statistical analyses for all studies were performed using unpaired, two-tailed Student's *t*-tests and one-way analysis of variance (ANOVA) for multiple group comparisons (GraphPad Prism 5, San Diego, CA, USA). *p*-values < 0.05 were considered statistically significant.

## SUPPLEMENTARY MATERIALS FIGURES


