# Supplementary material for: Co-localization of autophagy-related protein p62 with cancer stem cell marker dclk1 may hamper dclk1's elimination during colon cancer development and progression
Source: Oncotarget. 2019 Mar 22;10(24):2340–54. doi: 10.18632/oncotarget.26684 (PMC6481322; doi:10.18632/oncotarget.26684)
Supplement: Supplementary file 1 [file oncotarget-10-2340-s001.pdf]

Co-localization of autophagy-related protein p62 with Cancer Stem Cell marker Dclk1 may hamper Dclk1's elimination during colon cancer development and progression

SUPPLEMENTARY MATERIALS

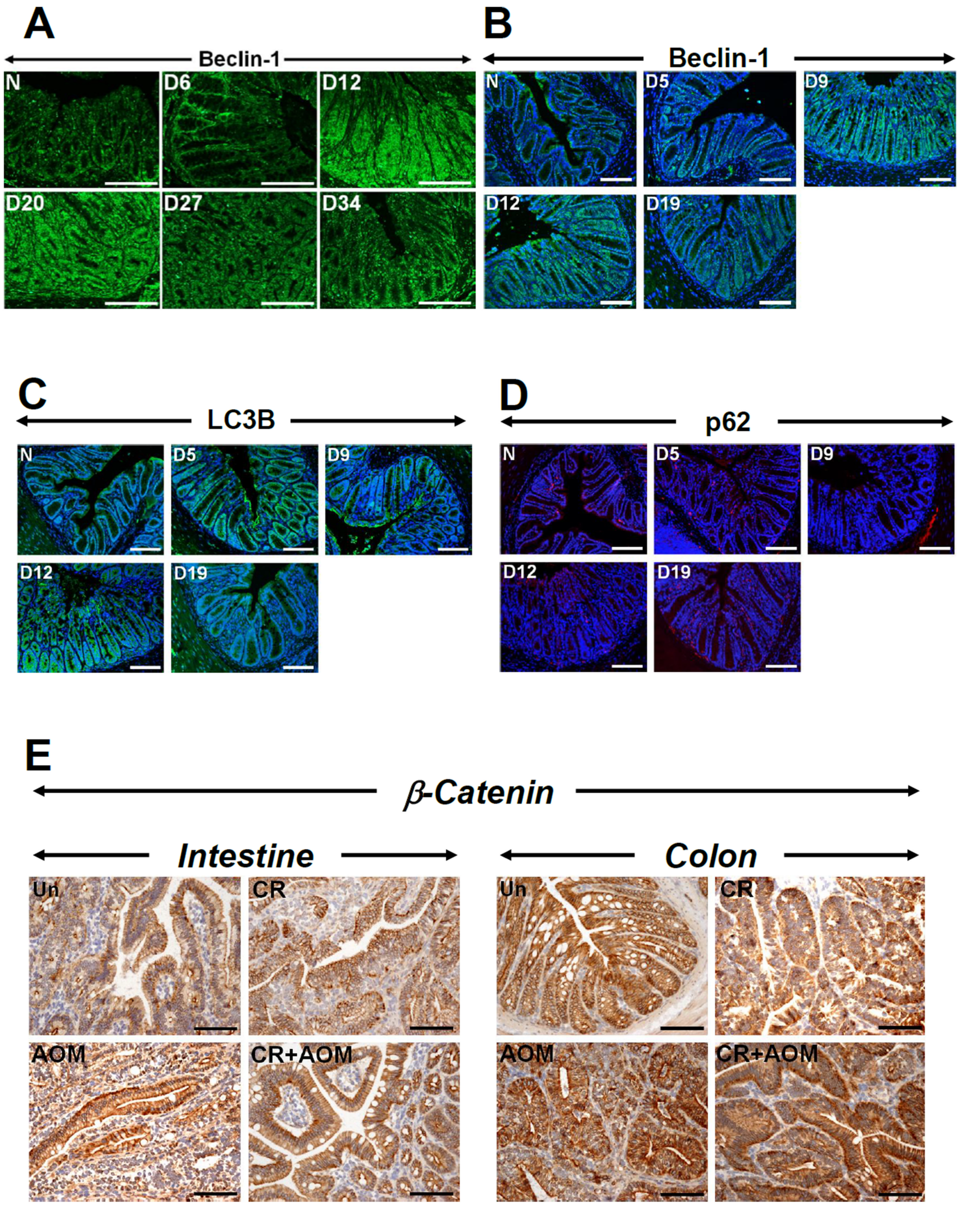

Supplementary Figure 1: Immunofluorescence (IF) images of Beclin1, LC3B and p62. (A) IF images of Beclin1 of the distal colons of NIH:Swiss uninfected (N) or mice infected with CR at days 6, 12, 20, 27 and 34. IF images of Beclin1 **(B)** LC3B **(C)** and p62 **(D)** of the distal colon of C57Bl/6 uninfected (N) or mice infected with CR at days 5, 9, 12 and 19. Images merged with DAPI (blue) showing nuclear staining. **(E)** Paraffin-embedded sections prepared from the intestinal or colonic tumors isolated from uninfected (N) or CR infected Apc1638N/+ mice were subjected to immunohistochemistry (IHC) staining for β -catenin. Scale bars = 150-250 μ m; n = 3 independent experiments.

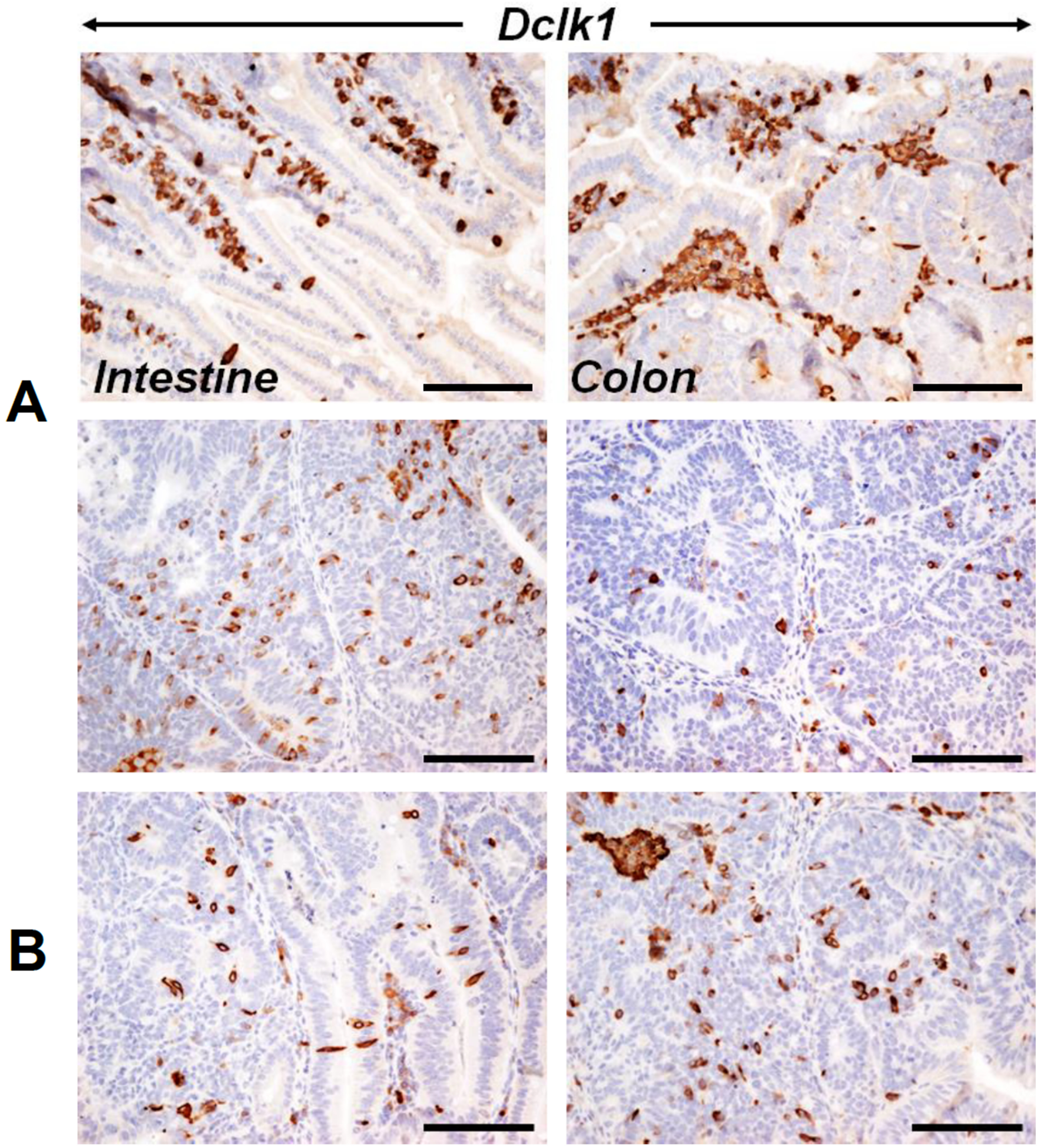

Supplementary Figure 2: Paraffin-embedded sections prepared from the intestinal or colonic tumors isolated from APC$^{min/+}$ mice were subjected to IHC staining with anti-Dclk1 antibody **(A)** & **(B)**. Scale bars = 450 μ m.

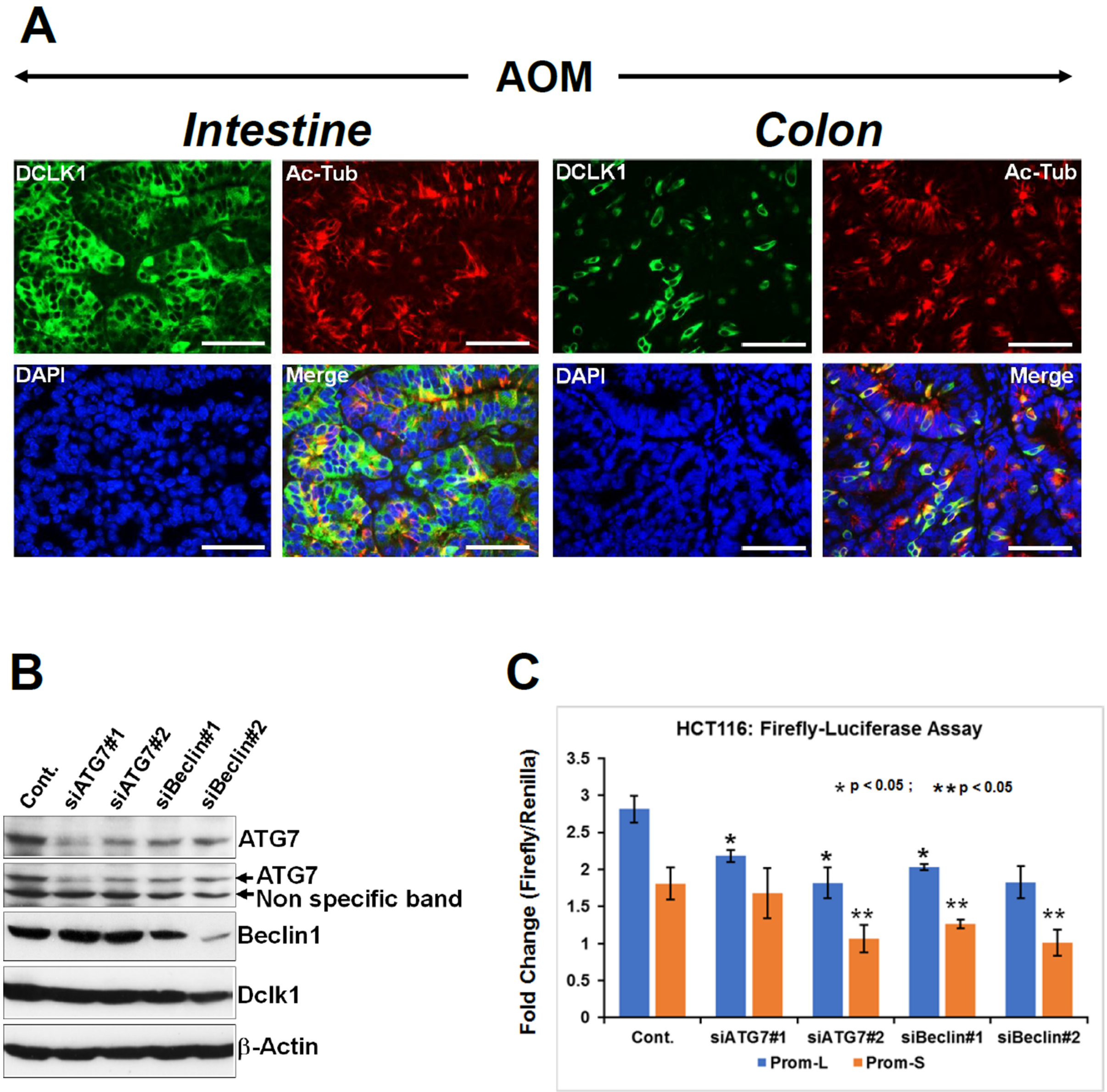

Supplementary Figure 3: Paraffin-embedded sections prepared from the intestinal or colonic tumors isolated from uninfected (N) or CR infected Apc1638N/+ mice were subjected to IF staining for DCLK1 and Ac-Tubulin. DAPI was used for nuclear stain **(A)**. Scale bars = 150-250 μ m; n = 3 independent experiments. **(B)** Western blot analyses of HCT116 cells treated with siRNAs shown above and probed with different antibodies shown on right. β -Actin was used as a loading control. **(C)** Results of Luciferase reporter assay of HCT116 cells treated as above and transfected with long (Prom-L) and short (Prom-S) reporter constructs.

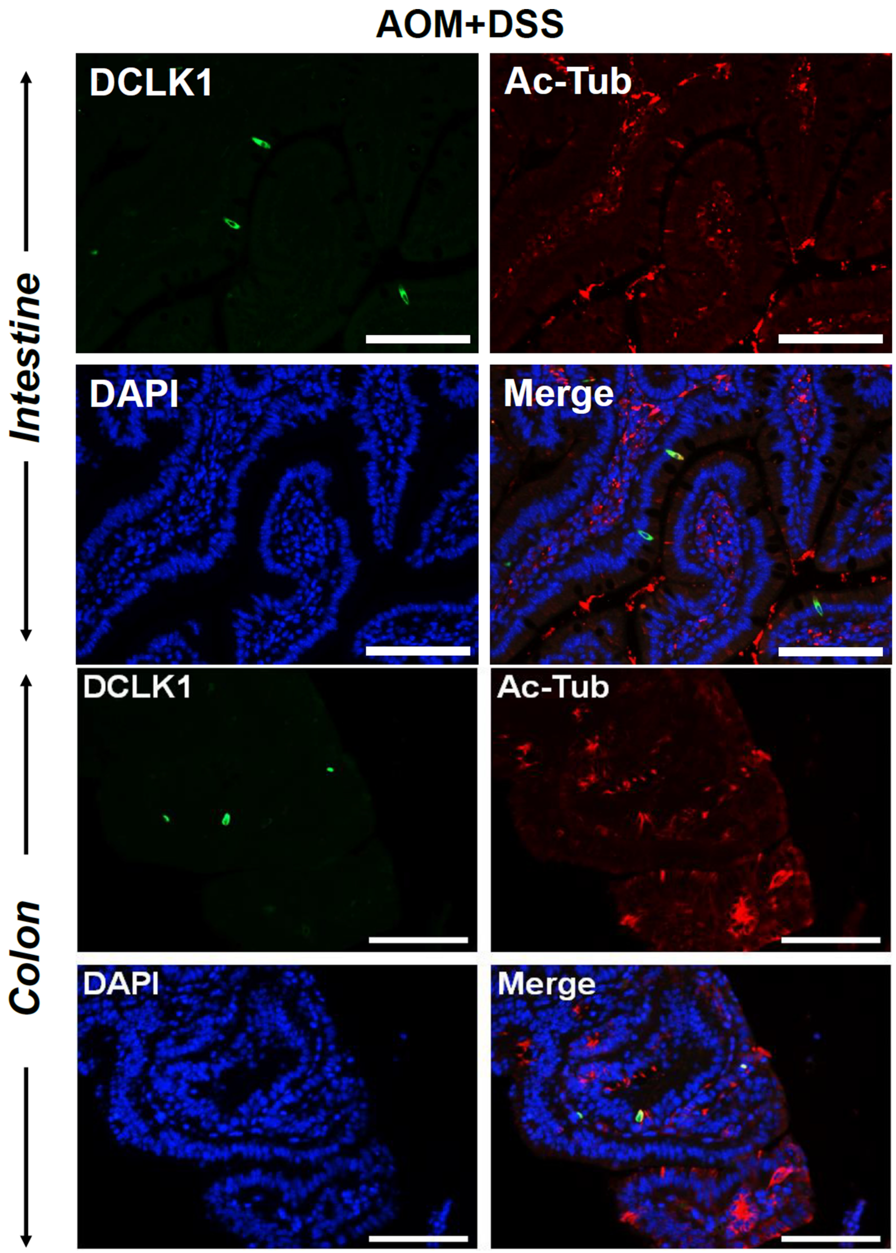

Supplementary Figure 4: Paraffin-embedded sections from intestinal or colonic tumors from AOM+DSS treated Apc1638N/+ mice were co-stained with Dclk1 and Ac-Tubulin. DAPI was used as nuclear stain. Scale bars = 450 μ m.

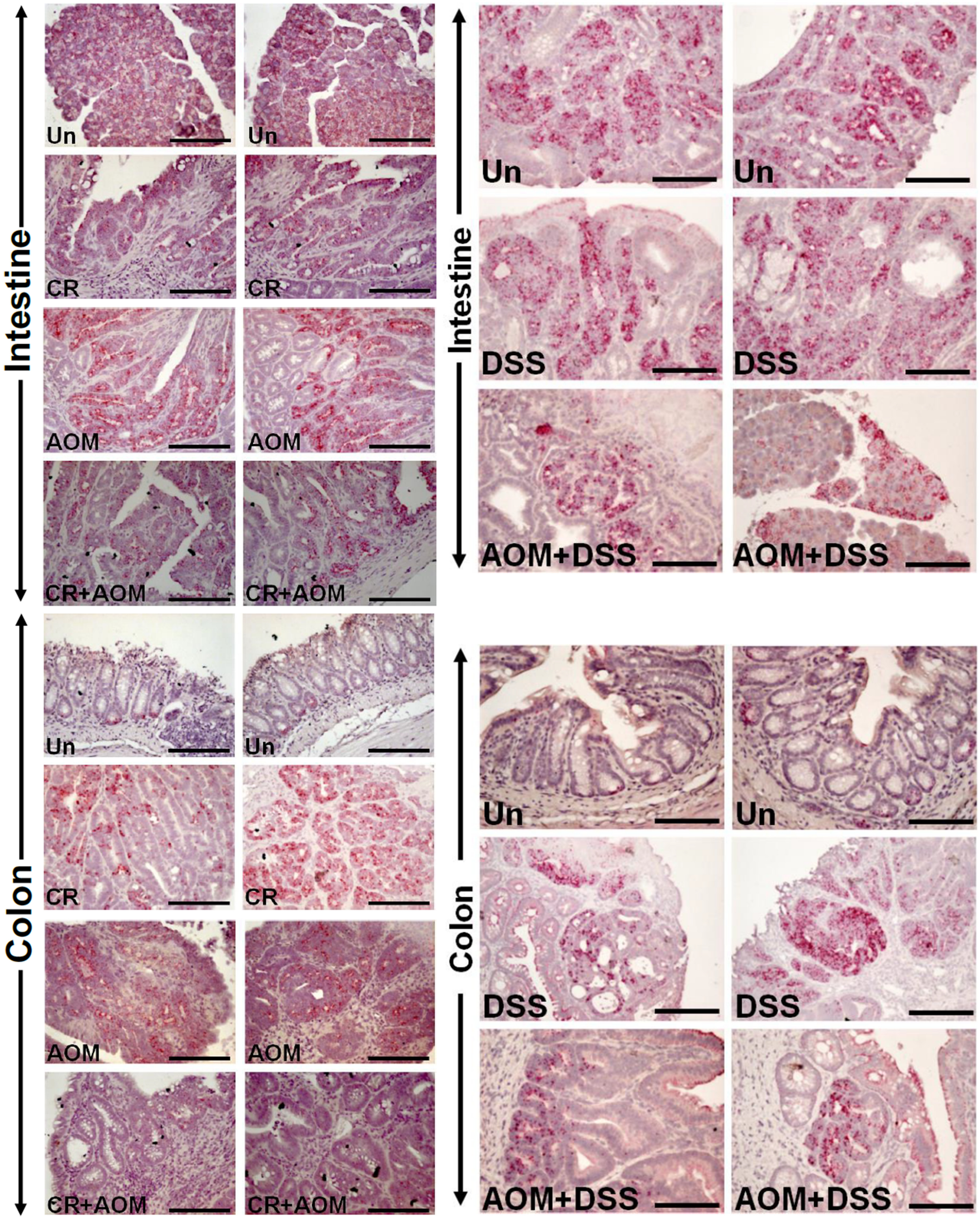

Supplementary Figure 5: Paraffin-embedded sections prepared from the intestinal or colonic tumors isolated from uninfected (N) or CR infected Apc1638N/+ mice were subjected to *in situ* hybridization (ISH) staining for Lgr5. Scale bars = 150-250 μ m.

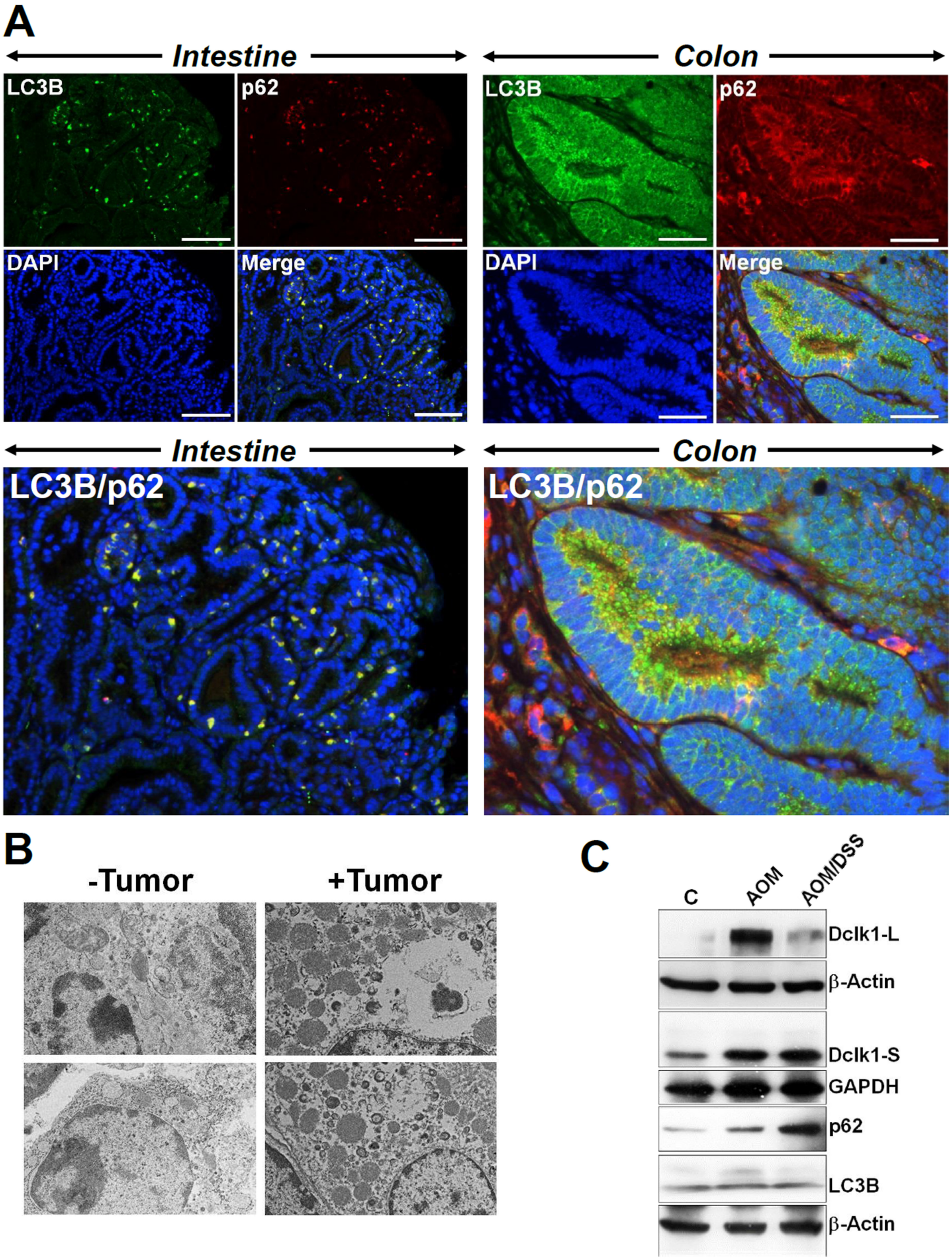

Supplementary Figure 6: **(A)** IF co-staining of LC3B and p62 on tissue sections as above. DAPI was for nuclear satin. **(B)** Electron microscopy in the normal (-Tumor) and tumor (+Tumor) tissue sections. **(C)** Western blot analyses of tissues as shown C as control, AOM and AOM/DSS treated and probed with antibodies shown on right. Scale bars = 450 μ m.

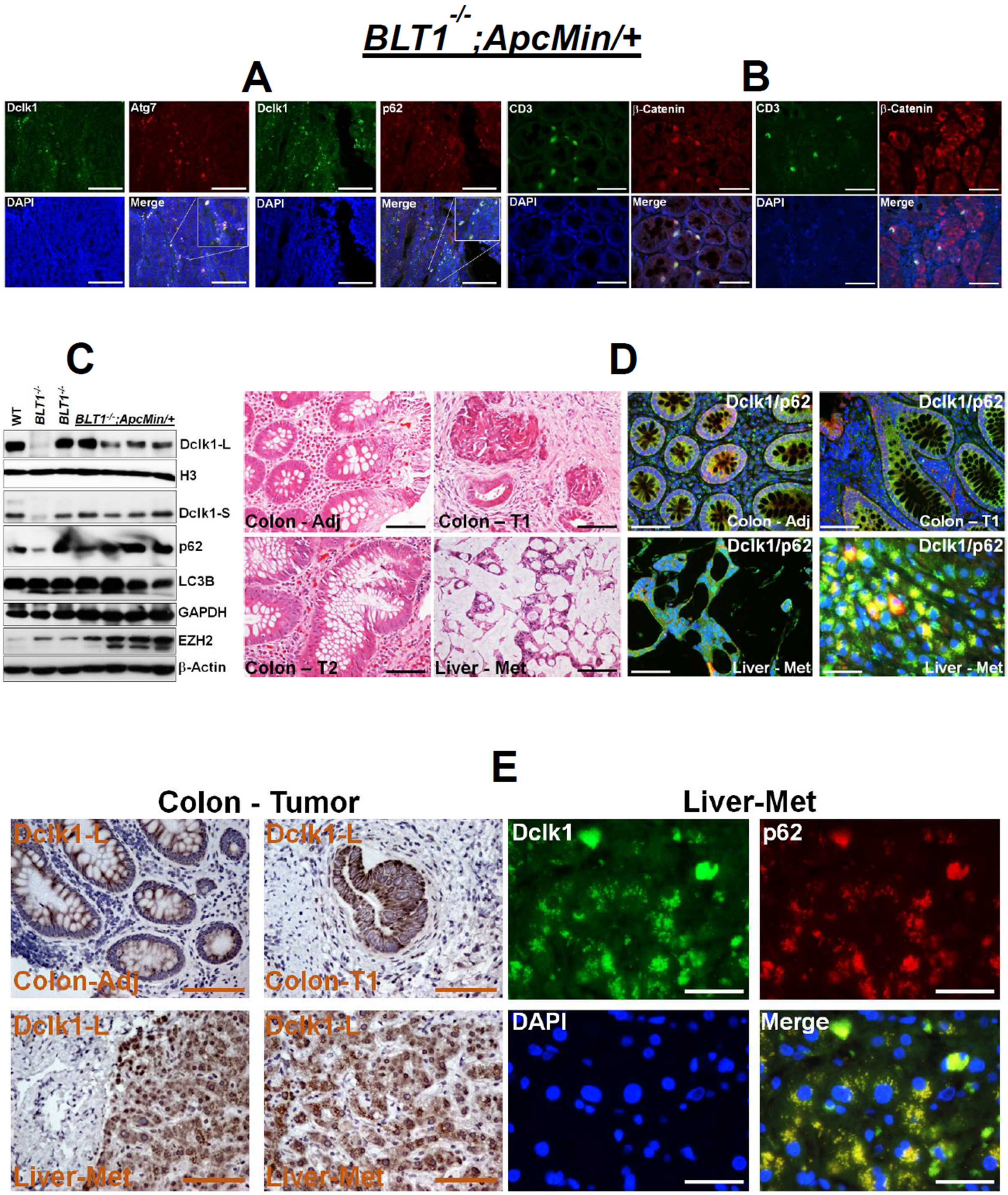

Supplementary Figure 7: **(A)** Dclk1 and its co-localization with Atg7 and p62. **(B)** CD3 and its co-localization with β -catenin In BLT1$^{-/-}$;Apc$^{Min/+}$ mice. DAPI was to nuclear staining. **(C)** Western blot analyses of different tissues as shown above and probed with various antibodies shown on right. **(D)** Hematoxylin and eosin (H&E) staining and Dclk1/p62 co-staining of surgical samples from a patient with multiple tumors in the colon and metastasis to the liver. **(E)** IHC staining of DCLK1 of the above samples and Dclk1/p62 co-staining of liver-metastasis. Scale bars = 150-250 μ m.
